# Effects of Isoflavone Supplementation on the Response to Medroxyprogesterone in Premenopausal Women with Nonatypical Endometrial Hyperplasia: A Randomized, Double-Blind, Placebo-Controlled Trial

**DOI:** 10.1155/2022/1263544

**Published:** 2022-11-24

**Authors:** Zahra Vahedpour, Homa Boroumand, Shirin Tabatabaee Anaraki, Zohre Tabasi, Hossein Motedayyen, Hossein Akbari, Fariba Raygan, Vahidreza Ostadmohammadi

**Affiliations:** ^1^Department of Gynecology and Obstetrics, Faculty of Medicine, Kashan University of Medical Sciences, Kashan, Iran; ^2^Infectious Diseases Research Center, Kashan University of Medical Sciences, Kashan, Iran; ^3^Autoimmune Research Center, Kashan University of Medical Sciences, Kashan, Iran; ^4^Department of Biostatistics and Epidemiology, Faculty of Public Health, Kashan University of Medical Sciences, Kashan, Iran; ^5^Department of Cardiology, Faculty of Medicine, Kashan University of Medical Sciences, Kashan, Iran

## Abstract

**Objective:**

The purpose of this study was to evaluate the impact of isoflavone supplementation compared with placebo on endometrial histology and serum estradiol levels in premenopausal women with nonatypical endometrial hyperplasia.

**Materials and Methods:**

The present double-blindplacebo-controlled clinical trial was conducted on 100 women with nonatypical endometrial hyperplasia in the age range of 30 to 45 years. Participants were randomly assigned to receive 50 mg of isoflavone (*n* = 50) or placebos (*n* = 50) daily for three months. Both groups received the standard treatment of nonatypical endometrial hyperplasia. Endometrial biopsy and blood samples were taken at the baseline and three months after the intervention. The incidence of drug side effects was assessed as well.

**Results:**

After three months, 88.4% of isoflavone-administered subjects had a significant histological improvement compared to 68.9% subjects in the placebo group (*P*=0.02). There were no significant differences between the two groups in the changes of serum estradiol levels and the incidence of drug side effects.

**Conclusion:**

The findings of the present study demonstrated that the coadministration of 50 mg of isoflavones and medroxyprogesterone acetate increases the treatment efficacy in women with nonatypical endometrial hyperplasia. *Clinical Trial Registration*. This trial was registered on the Iranian website for clinical trial registration (https://www.irct.ir/trial/53553).

## 1. Introduction

Endometrial cancer is the most prevalent malignancy of the female reproductive system in developed countries [1]. Endometrial hyperplasia is a precursor lesion for adenocarcinoma of the endometrium, which accounts for the majority of endometrial cancer [2]. Chronic exposure to unopposed estrogen is the main risk factor for the development of endometrial hyperplasia [3]. Abnormal endometrial histology such as endometrial hyperplasia is relatively common (about 14%) among premenopausal women with irregular menses [4]. The risk of developing endometrial cancer following endometrial hyperplasia depends on the presence of atypia and its severity [5]. Endometrial hyperplasia without atypia regresses following curettage or progestin treatment and has a lower risk of progression to adenocarcinoma [6], but lesions with atypia often do not regress and may be resistant to repeated curettage or long-term treatment with progestin [7]. Previous studies have reported that surgery is the best strategy for treating endometrial hyperplasia with atypia, but the tendency to find alternative medical treatments for surgery with lower complications and higher efficacy is currently increasing [8]. There are many progesterone-containing medications for the treatment of endometrial hyperplasia such as medroxyprogesterone, megestrol, and norethindrone acetate [9].

Recently, herbal supplements such as soy-derived isoflavone have been widely investigated for the treatment of nonatypical endometrial hyperplasia [10]. Genistein, a soy-derived isoflavone, was reported to be one of the new agents for the treatment of endometrial hyperplasia, which was more effective than norethindrone acetate [11]. Isoflavone supplementation may prevent endometrial cancer due to its beneficial effects on cell proliferation, apoptosis, and progesterone receptors [12, 13]. Isoflavones have both estrogenic and antiestrogenic effects, which can be used for the treatment of breast and prostate cancers, symptomatic amelioration of postmenopausal women, and osteoporosis [14]. Shukla et al. [15] showed genistein at a daily dosage of 54 mg can decrease the local synthesis of estrogen and may inhibit progression of endometrial hyperplasia to endometrial cancer. Moreover, Steinberg et al. [16] revealed that isoflavone supplementation for two years has a safety profile without any serious adverse effects in women.

Considering the high prevalence of endometrial hyperplasia and possible drug side effects of hormone-containing medications, administration of herbal medicines along with routine treatment may enhance treatment efficacy. Therefore, this study was performed to evaluate the effects of isoflavone supplementation compared with placebo in the treatment of women with nonatypical endometrial hyperplasia.

## 2. Materials and Methods

### 2.1. Participants and Ethics Statement

The present investigation has been registered on the Iranian website for registration of clinical trials with the special ID of IRCT20200531047614N3 (https://www.irct.ir/trial/53553) after obtaining approval by the Research Ethics Committee of the Kashan University of Medical Sciences (KAUMS) and following the Declaration of Helsinki and Good Clinical Practice guidelines. This clinical trial was performed at the Gynecology Clinic of Shahid Beheshti Hospital (Kashan, Iran) between May 2020 and July 2021. After a face-to-face meeting, we explained the objective and methods of the study to patients, and written informed consent was obtained from all women.

### 2.2. Inclusion/Exclusion Criteria

#### 2.2.1. Inclusion Criteria

The inclusion criteria were as follows:

1. Women who were diagnosed with nonatypical endometrial hyperplasia
2. Aged 30 to 45 years old

#### 2.2.2. Exclusion Criteria

The exclusion criteria were as follows:

1. Unwillingness to cooperate
2. Receiving hormone therapy within 6 months prior to enrollment in the trial
3. A history of atypical hyperplasia
4. Menopausal women
5. Hypersensitivity to soybean products
6. Patients with focal endometrial lesions
7. Women with congenital uterine anomalies

### 2.3. Sample Size Calculation

We used a randomized clinical trial sample size calculation formula, where type one (alpha) and type two (beta) errors were 0.05 and 0.10 (power = 90%), respectively. The sample size was determined according to a study by Bitto et al. [11] to compare the effects of isoflavones and placebos on endometrial hyperplasia. In this trial, 42% of isoflavone-administered participants (*P*_1_=0.42) had a considerable improvement in symptoms compared to 12% of placebo-received subjects (*P*_2_=0.12). Using the formula, we needed 41 participants in each group; after allowing for 20% dropouts in each group, the final sample size was 50 subjects in each group.

### 2.4. Study Design

In this randomized, double-blind, placebo-controlled trial, after balanced block randomization, participants were allocated into two intervention groups. The randomization list was created from 1 to 100 by a random number generator website (https://stattrek.com/statistics/random-number-generator.aspx), and women were randomly assigned into two groups to receive either isoflavone (*n* = 50) or placebos (*n* = 50). The medical team, participants, and data assessors were blinded to treatment allocation. The patients in the experimental group received 50 mg/day isoflavone supplements (Goldaru Pharmaceutical Company, Tehran, Iran), and subjects in the control group (*n* = 50) received daily one placebo tablet (Goldaru Pharmaceutical Company, Tehran, Iran) for three months. Isoflavone supplements and placebos had similar packaging, color, shape, and size. Based on the information from the pharmaceutical company, an ultrasound-assisted extraction (UAE) method [17] was employed in the isolation of isoflavone from soybeans. Qualitative and quantitative analyses of isoflavones were determined by high-performance liquid chromatography (HPLC). Both groups received standard treatment for nonatypical endometrial hyperplasia with 10 mg of medroxyprogesterone acetate daily for two weeks per month for a three-month intervention period. To ensure that patients take tablets, while following up by phone, they were asked to bring the empty package of medication after the intervention.

### 2.5. Assessment of Outcomes

The primary outcome was the pathological response, but serum estradiol levels and drug side effects were considered secondary outcomes. After obtaining informed written consent, a checklist, including demographic characteristics and disease-related variables, was filled out. At the baseline and after the 3-month intervention period, blood samples (10 mL) were collected from each patient at the Shahid Beheshti Gynecology Clinic. Estradiol concentration was quantified using a solid-phase immunoassay (Roche Diagnostics, Milan, Italy). At the beginning and end of the third month, an endometrial biopsy was performed by a gynecologist (ZV). Samples were evaluated by the same pathologist initially and after the intervention. Women were instructed to report any probable side effects such as edema, weakness, anorexia, mastalgia, abdominal pain, and dizziness.

### 2.6. Statistical Analysis

The normality of data was determined by the Kolmogorov–Smirnov test. To find out significant changes in continuous variables between the two groups, we applied independent *t*-tests. Pearson Chi-square tests were utilized to compare categorical variables. An analysis of covariance (ANCOVA) was used to evaluate the impact of isoflavone supplementation compared with placebos on serum estradiol levels after adjusting for confounding parameters including age and baseline estradiol values. A *P* value less than 0.05 was considered statistically significant. All analyses were performed by using SPSS version 16 (Chicago, Illinois, USA).

## 3. Results

**Figure 1:**
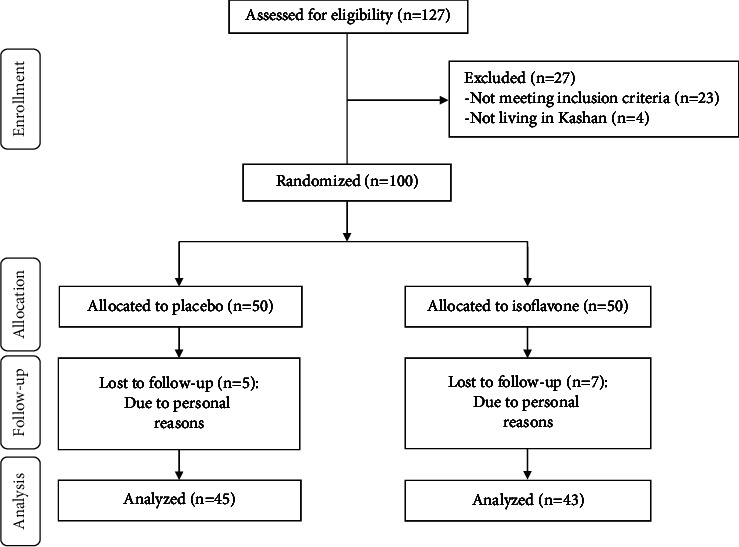
Summary of the patient flow diagram.

As shown in this study flow diagram (Figure 1), we enrolled 100 premenopausal women with nonatypical endometrial hyperplasia in this trial; however, 12 participants were excluded from the trial because of personal reasons. Finally, 88 participants (placebo (*n* = 45) and isoflavone (*n* = 43)) completed the trial.

Baseline characteristics of participants including height, age, weight, body mass index (BMI), systolic blood pressure and diastolic blood pressure, and the percentage of diabetes mellitus were not statistically different between the two groups (Table 1).

After three months, isoflavone and medroxyprogesterone coadministration significantly improved endometrial hyperplasia compared with placebo plus medroxyprogesterone. The efficacy of isoflavone plus medroxyprogesterone in the treatment of nonatypical endometrial hyperplasia was 88.4%, while placebo plus medroxyprogesterone treated 68.9% of patients, and this difference was statistically significant (*P*=0.02). Moreover, isoflavone supplementation for 3 months had no significant effect on serum estradiol levels compared with the placebo (Table 2).

Drug side effects while taking isoflavones and placebos were investigated in two groups of women with nonatypical endometrial hyperplasia. According to the results of the study, the incidence of decreased appetite, dizziness, edema, mastalgia, abdominal pain, and weakness was not statistically significant between the two groups (Table 3).

## 4. Discussion

To the best of our knowledge, this trial is the first investigation to evaluate the effects of isoflavone supplementation compared with the placebo on endometrial histology and serum estradiol levels in premenopausal women with nonatypical endometrial hyperplasia. Our study indicated that administration of isoflavone (50 mg/day for 3 months) to women with nonatypical endometrial hyperplasia significantly improved endometrial histology, but it had no effect on serum estradiol levels. In the present trial, 88.4% of isoflavone-administered subjects had a significant histological improvement compared to 68.9% subjects in the placebo group.

Isoflavone is a phytoestrogen that is found in plants such as soybeans [18]. The human estrogen receptor (ER) exists as two subtypes (ER-alpha and ER-beta) [19]. ER-beta has an inhibitory effect on ER-alpha and is the dominant receptor type in the brain and cardiovascular system [4]. The dominant receptor type in the uterus is ER-alpha [20]. The affinity of phytoestrogen to bind to ER-beta is greater than ER-alpha [21]. On the other hand, isoflavone (low dose) can bind to ER-alpha in the endometrium of the uterus and occupy the active site of the receptor and prevent the binding of endogenous estrogen, which is more efficient than isoflavones, and thus acts as a partial agonist [22]. Furthermore, isoflavone can prevent endometrial hyperplasia and cancer due to its effects on cell proliferation, apoptosis, and progesterone receptors [23]. The prolonged use of high-dose isoflavone (≥150 mg) can act as estrogen agonists, with a greater effect on the alpha receptor, and cause the proliferative effects of estrogen on the uterine endometrium [24]. Therefore, soybean-derived isoflavones at a dose of 150 mg may cause endometrial hyperplasia, and at a dosage of 50 mg, they can reduce hyperplasia and exert protective effects on the endometrium and decrease the progression of lesions to endometrial cancer [25]. Various studies have evaluated isoflavone supplementation with different doses and duration of intervention in women. Unfer et al. [26] investigated the effects of long-term (5 years) use of isoflavone on endometrial histology in healthy postmenopausal women. In this study, women were assigned to two groups receiving 150 mg of isoflavones or placebos daily, and the endometrial biopsy was performed at the baseline of the study and five years later. The findings of this study revealed that the incidence rate of hyperplasia in the isoflavone group (3.37%) was significantly higher than that in the placebo group (0%). In addition, endometrial atrophy was observed in 71% of women receiving isoflavones and 80% of women taking placebos at the end of the intervention. No malignancy occurred in the two groups. Murray et al. [27] demonstrated that isoflavone supplementation (120 mg daily) for six months compared with placebos in menopausal women treated with exogenous estrogen replacement therapy had no significant impact on the incidence rate of endometrial hyperplasia. Bitto et al. [11] compared the effects of isoflavone and norethindrone acetate administration on endometrial histology, estrogen and progesterone receptor gene expression, and serum levels of sex hormones in 56 women with nonatypical endometrial hyperplasia. It has been shown that after 6 months, 42% of isoflavone-administered individuals (at a daily dosage of 54 mg) had a considerable improvement in symptoms compared to 12% subjects in the placebo group. Changes in hormone profiles were not statistically different between the two groups. The genes expression related to estrogen receptors (ER-alpha and ER-beta) significantly decreased in the isoflavone group. In a study by Quaas et al. [28] on 350 postmenopausal women aged 45–92 years, it was found that daily administration of 154 mg isoflavone for three years compared with placebos had no significant effect on the incidence rate of endometrial hyperplasia, endometrial thickness, and the incidence rate of malignancy. The main possible mechanisms of isoflavone in the recovery of nonatypical endometrial hyperplasia include decreasing mRNA expression of ER-alpha [29], matrix metalloproteinases-26 [30], increasing gene expression of the progesterone receptor [31], and being a partial agonist for ER-alpha [32]. Based on a recent meta-analysis study, low-dose isoflavone (dose <100 mg/day) has a safety profile without any serious side effects. Only minimal adverse effects such as abdominal pain, diarrhea, myalgia, and headache have been reported [33].

The limitations of this trial include the short period of intervention, relatively small sample size, and single-center design. Furthermore, due to inappropriate funding, there was no chance to evaluate other hormonal parameters such as serum progesterone levels, LH and FSH concentrations, and gene expression related to estrogen and progesterone receptors at the baseline and three months after the intervention.

## 5. Conclusion

Overall, the coadministration of 50 mg of isoflavone and medroxyprogesterone acetate for three months increases the efficacy of treatment in premenopausal women with nonatypical endometrial hyperplasia.

## Figures and Tables

**Table 1:** General characteristics of study participants.

	Placebo group (*n* = 45)	Isoflavone group (*n* = 43)	*P* ^a^
Age (y)	37.2 ± 4.2	37.7 ± 4.1	0.59
Height (m)	158.7 ± 9.0	161.0 ± 9.9	0.24
Weight at the study baseline (kg)	75.4 ± 10.1	77.7 ± 9.2	0.27
Weight at the end of the trial (kg)	75.7 ± 10.2	77.9 ± 9.6	0.29
BMI at the study baseline (kg/m^2^)	30.1 ± 4.7	30.2 ± 4.3	0.96
BMI at the end of the trial (kg/m^2^)	30.2 ± 4.8	30.3 ± 4.3	0.99
SBP (mmHg)	124.3 ± 10.9	122.2 ± 10.0	0.34
DBP (mmHg)	75.5 ± 7.2	75.7 ± 5.3	0.91
Diabetes mellitus	5 (11.1%)	6 (14%)	0.68^*∗*^

BMI, body mass index; DBP, diastolic blood pressure; SBP, systolic blood pressure. Data are presented as a mean ± SD or numbers (%). ^a^Obtained from the independent samples *t*-test. ^*∗*^Obtained from the chi-square test.

**Table 2:** Endometrial histology and serum estradiol levels at the baseline and after the 3-month intervention period in women with nonatypical endometrial hyperplasia that received either isoflavone or the placebo.

Variables	Placebo group (*n* = 45)		Isoflavone group (*n* = 43)		
	Baseline	Month 3	Baseline	Month 3	*P*
Endometrial histology					**0.02** ^*∗*^
Normal	0	31 (68.9)	0	38 (88.4)	
Hyperplasia	45 (100)	14 (31.1)	43 (100)	5 (11.6)	
Estradiol (pg/ml)	89.1 ± 21.8	81.2 ± 20.8	85.2 ± 21.2	75.3 ± 19.6	0.26^†^

Data are presented as a mean ± SD or numbers (%). ^*∗*^Obtained from the chi-square test. ^†^Obtained from the analysis of covariance (adjusted for the age and baseline estradiol value).

**Table 3:** Drug side effects in two intervention groups.

	Placebo group (*n* = 45)	Isoflavone group (*n* = 43)	*P* ^a^
Anorexia	7 (15.6)	5 (11.6)	0.59
Dizziness	3 (6.7)	2 (4.7)	0.68
Edema	1 (2.2)	1 (2.2)	0.97
Mastalgia	5 (11.1)	3 (7)	0.71
Abdominal pain	1 (2.2)	0	0.32
Weakness	4 (8.9)	6 (14)	0.45

Data are presented as numbers (%). ^a^Obtained from the chi-square test.

## Data Availability

The datasets analyzed during the current study are available from the corresponding authors on reasonable request.

## Abbreviations

BMI: Body mass index
DBP: Diastolic blood pressure
ER: Estrogen receptor
FSH: Follicle-stimulating hormone
LH: Luteinizing hormone
SBP: Systolic blood pressure.
